# Unraveling the molecular landscape of congenital pseudoarthrosis of the tibia: insights from a comprehensive analysis of 159 probands

**DOI:** 10.1186/s13023-025-03759-4

**Published:** 2025-06-03

**Authors:** Rui Wang, Yu Zheng, Ge Yang, Zhenchao Xu, Yaoxi Liu, Weihua Zhao, Hua Wang, Haibo Mei, Guanghui Zhu

**Affiliations:** 1 Department of Medical Administration, The Affiliated Children’s Hospital of Xiangya School of Medicine (Hunan Children’s Hospital), Central South University, Changsha, 410007 Hunan People’s Republic of China; 2 Department of Medical Genetics, The Affiliated Children’s Hospital of Xiangya School of Medicine (Hunan Children’s Hospital), Clinical Medical Research Center for Hereditary Birth Defects and Rare Diseases In Hunan Province, Central South University, 86 Ziyuan Road, Changsha, Hunan China; 3 Department of Pediatric Orthopedics, Hunan Provincial Key Laboratory of Pediatric Orthopedics, The Affiliated Children’s Hospital of Xiangya School of Medicine (Hunan Children’s Hospital), Central South University, Changsha, 410007 Hunan People’s Republic of China; 4 Furong Laboratory, Changsha, Hunan China; 5 MOE Key Lab of Rare Pediatric Diseases, University of South China, Hengyang, 421001 Hunan People’s Republic of China; 6 The School of Pediatrics, University of South China, Hengyang, 421001 Hunan People’s Republic of China

**Keywords:** Congenital pseudarthrosis of the tibia, Whole exome sequencing, Genetic mutations, *NF1*

## Abstract

**Background:**

Congenital pseudarthrosis of the tibia (CPT, HP:0009736), commonly known as bowing of the tibia, is a rare congenital tibia malformation characterized by spontaneous tibial fractures and difficulty in reunion after tibial fractures during early childhood, with a prevalence between 1/250,000 and 1/140,000. While 80%–84% of CPT cases present with neurofibromatosis type 1, caused by the variations in the *NF1* gene, the underlying cause of CPT remains unclear. Considering its congenital nature and the low prevalence, we hypothesized that the rare genomic protein-damaging variations may contribute to CPT.

**Results:**

In this study, we conducted whole exome sequencing on 159 patients with CPT and found loss-of-function (LoF) excesses in the 159 patient cases compared to 208 healthy controls from the 1000 Genomes Project. The LoF variant types primarily included stop-gained and frameshift variants, both present in 97% of the 159 patients with CPT, as well as splice-changing variants, which were found in 78% of these patients. Rare LoF variations in osteocyte-related pathways, such as ossification, were identified in 112 of the 159 CPT cases (70.4%). The top seven genes carrying rare protein-damaging variants that might be related to CPT are *NF1*, *GLI3*, *MRC2*, *PTH1R*, *RYR1*, *NPR2* and *ITGA11.*

**Conclusions:**

These findings shed light on novel genetic mutations and osteocyte transcriptome-related molecular pathways involved in CPT, providing a new framework for understanding the genetic regulation of CPT pathology and suggesting potential directions to further elucidate its pathogenesis.

**Supplementary Information:**

The online version contains supplementary material available at 10.1186/s13023-025-03759-4.

## Introduction

Congenital pseudarthrosis of the tibia (CPT) is a rare orthopedic disease with an incidence of 1/140,000 to 1/250,000 live births [1]. It is characterized by the pseudarthrosis or pathological fractures of the anterolateral part of the tibia in early life, resulting in bowing and narrowing of the medullary canal, or the presence of a cyst. This condition poses a significant surgical challenge due to recurrent fractures and the inability to achieve bone union [2]. Congenital anterolateral bowing of the tibia is generally considered a precursor of CPT and is commonly associated with neurofibromatosis type 1 (*NF1*), a common autosomal dominant genetic disorder [3]. Previous research has reported that 80%–84% of CPT cases present with *NF1* (*NF1*-CPT) in the epidemiology and *NF1* gene mutations have been linked to CPT in the genomics [4–7]. The double inactivation model has been proposed as a hypothesis to explain these associations [8–11]. However, other researchers have suggested that the molecular pathogenesis of *NF1*-CPT may not be entirely explained by second mutations or loss of heterozygosity of *NF1* [7, 12, 13].

Recent investigations have shed light on the molecular basis of CPT. *NF1* is involved in bone remodeling and mineralization [14]. Inhibited bone formation and stimulated bone resorption have been reported in patients with CPT who harbor *NF1* mutations [15]. Furthermore, the involvement of FGFR3 signaling in cartilage-to-bone transformation for bone repair [16] and the contribution of vasoactive intestinal peptide through ERK and NF-κB signal pathways have been implicated in CPT [17]. Differentially expressed proteins found in serum-derived exosomes of patients with CPT have been shown to inhibit bone formation and stimulate bone resorption [15], while differentially expressed proteins in the tibia periosteum tissues are mainly involved in cell matrix assembly, cell adhesion, AKT-PI3K signal pathway activation, and vascular agglutination [18]. These conclusions indicate that the intricate biological processes and molecular regulation pathways associated with bone homeostasis potentially contribute to CPT [19, 20]. However, a comprehensive understanding of the etiology of CPT is still elusive due to its rarity and limited samples in previous studies.

Genetic research on other complex bone disorders highlighted that mutations in genes with highly enriched expression in osteocytes could cause rare bone and mineral disorders. For example, autosomal recessive inactivating mutations in *SOST*, which encodes the Wnt-antagonist sclerostin, result in the high bone mass disorder sclerosteosis type 1 (OMIM: 269,500) [21]. Deletion of an SOST regulatory element causes van Buchem disease (OMIM 239100) and inactivating mutations in *DMP1*, the gene encoding dentin matrix acidic phosphoprotein 1, cause autosomal recessive hypophosphataemia (OMIM: 241,520). These findings suggest that rare protein-damaging variations (PDVs) may play important roles in the genetic etiology of CPT.

PDVs, particularly loss of function (LoF) mutations, have been identified as key contributors to congenital developmental diseases characterized by low prevalence and early onset. In light of this, we hypothesized that CPT could also be explained, at least partially, by rare PDV variants with incomplete penetrance. To test this hypothesis, we conducted whole exome sequencing (WES) on a substantial cohort of 159 individuals affected by idiopathic CPT. This approach is widely used in investigating severe pediatric developmental disorders with low prevalence and complex genetic architecture, and has been successful in elucidating the genetic basis of other neurodevelopmental disorders, including epilepsy [22–26], craniosynostosis [27–29], autism [30–38] and congenital heart disease [39–44].

Here, we present our comprehensive genetic analysis, aiming to uncover the complex genetic architecture and molecular mechanism underlying CPT. Our findings have the potential to enhance the understanding of CPT pathogenesis and provide valuable guidance for the development of novel therapeutic approaches targeting the identified genetic factors.

## Method

### Patients

The inclusion criteria for cases were children under 18 years old with a clinical diagnosis of CPT, regardless of surgical history. Exclusion criteria included: (1) tibial pseudarthrosis caused by trauma, tumors, or other factors, and (2) patients with osteofibrous dysplasia. The diagnosis of CPT was based on patients experiencing a tibial fracture after minor trauma with a subsequent inability to achieve bone union through casting or orthosis, and X-rays showing anterolateral bowing, medullary canal narrowing, a cyst, or non-union of the tibia. A consecutive cohort of 159 Chinese CPT cases (99 males and 60 females) was enrolled in this study (Supplementary Table 1). The average age of the enrolled patients was 42.7 months (range: 1 month to 13.3 years). These patients were admitted into the Department of Orthopedics of Hunan Children’s Hospital from 2016 to 2020. For WES, peripheral blood samples were collected from all 159 CPT cases for DNA extraction and sequencing, of which 75 cases had been previously reported in 2019 [7]. This study was approved by the Ethics Committee of Hunan Children’s Hospital, and informed consent was obtained from all patients.

### WES variation calling and filtering

Genomic DNA from peripheral blood was extracted using the standard phenol–chloroform method. The DNA of all 159 CPT cases was fragmented and the exome was captured using the Agilent SureSelect Human All Exon V6 kit. The captured DNA was sequenced with 100 bp pair-end reads using the BGISEQ2000 platform, following the manufacturer’s instructions. Each sample yielded an average of 13.3 Gb of raw data with a mean of 136 times by independent reads, with 98.73% reading 20 or more times. The coverage ratio was 99.84% for the captured target region of 60.46 M. Over 98% (average: 92.9%) of the bases had a quality score of > 30.

The sequencing libraries were constructed using MGIEasy Universal DNA Library Prep Set (MGI), and the constructed libraries were captured using the MGIEasy Exome Capture V4 Probe (MGI) according to the manufacturer’s protocol. The hybridization capture products were subjected to post-capture amplification and then circular DNA was generated by the splint oligo ligation. The DNA nanoball libraries were generated by rolling circle amplification of circularized DNA libraries and were sequenced on the MGISEQ-2000 sequencer platform (MGI) in a strategy of paired-end 100 bp plus 10 bp (index) with an average depth of ≥ 180-fold. The process of bioinformatics analysis, including data filtering, alignment, mutation detection, and result annotation, was performed as previously described [45, 46]. Briefly, raw reads were filtered by SOAPnuke, and clean reads were mapped to the human reference genome GRCh37 (hg19) using the BWA-mem algorithm (BWA). Single nucleotide variants and insertion or deletion variants were called using the Genome Analysis Toolkit (GATK) and annotated by an in-house pipeline. For copy number variant (CNV) detection, exonic CNVs and large CNVs (> 1 M) were analyzed using ExomeDepth and CNVkit, respectively. Pathogenicity predictions were conducted by an in-house pipeline, which was implemented following the American College of Medical Genetics and Genomics and the Clinical Genome Resource.

For the variation calling, the raw reads were filtered by SOAPnuke [47] and then mapped to the human genome reference (UCSC/GRCh37/hg19) using the Burrows-Wheeler Aligner (BWA-MEM, version 0.7.10) [48]. Variants were called using the Genome Analysis Tool Kit (GATK, version 4.1) [49]. To filter out false-positive genotypes, we applied strict criteria to the WES variations. A genotype was marked as missing for any patient if it met any of the following three criteria: (1) the genotype quality was less than 30; (2) the read depth was less than 20; (3) the allele balance for a heterozygous genotype was less than 0.3 or greater than 0.7. Additionally, the variations with the missing rate > 0.8 in our 159 CPT cases were removed and all insertions/deletions with allele lengths greater than 10 bp were removed. Furthermore, the Variant Effect Predictor [50] was used to annotate and classify all the variants. Subsequently, for population frequency filtering, PDVs were removed if their frequency in the public database gnomAD (https://gnomad.broadinstitute.org/) or ExAC (http://exac.broadinstitute.org) was greater than 0.1%. For PDVs not registered in gnomAD and ExAC, those with a minor allele frequency greater than 0.01 also were removed. Finally, for the pathogenicity filtering, we retained pathogenic missense variations predicted by AlphaMissense and excluded variants with a CADD score less than 20 or a LoFtool score greater than 0.6 [51–53].

### LoF excess test for pathway gene sets

After obtaining the variant call format file of LoF variations, the software rvtest [54] was used to test the LoF excess for each gene set from the biological processes or KEGG pathways. The burden test was performed using Clade model C, with 208 Han Chinese individuals from the 1000 Genomes Project serving as the control population against the 159 patients with CPT. The variant call format files containing the variation information of both the 159 patients and the 208 control individuals are available upon request. The genes corresponding to the biological processes were obtained from http://amigo.geneontology.org/amigo/term/GO:0008150, with the organism selected as Homo sapiens. The genes of KEGG pathways were downloaded from https://www.genome.jp/kegg/.

## Results

### Rare singleton PDVs significantly enriched in 159 CPT cases

The WES of 159 Han Chinese CPT cases detected a total of 286,945 single nucleotide polymorphisms and 27,807 insertions/deletions. After removing false-positive genotypes and the non-pathogenic variations, there were 3043 rare singleton PDVs (SPDVs) in 2409 genes, including 1117 LoF and 1926 missense variations, which were considered potential contributors to CPT given its very low prevalence. The LoF variants mainly included stop-gained, frameshift and splice-changing variants.

To investigate enriched genes and potential genetic defects associated with CPT, we performed case–control association analyses at the variant and gene levels against 208 Han Chinese individuals from the 1000 Genomes Project. We found 3727 SPDVs significantly enriched in each consequence of variations in the 159 cases (Table 1). Stop-gained variants (OR = 5.92) and frameshift variants (OR = 14) were the two most frequent types found in the 159 CPT patients, each comprising 97% (154/159). Splice-changing variants followed, with 78% (124/159, OR = 20.67) having splice donor changes and 75% (119/159, OR = 10.82) having splice acceptor changes (Table 1).

**Table 1:** Rare LoF and missense variations are significantly enriched in 159 CPT patients against 208 control individuals

Var.type	Var.Num	Case.Num	Ctrl.Num	Case%	Ctrl%	OR	chisq.test
frameshift_variant	570	154	11	97	5	14	1.45E–67
stop_lost	17	17	0	11	0	17	2.19E–05
splice_acceptor_variant	189	119	11	75	5	10.82	1.09E–42
splice_donor_variant	196	124	6	78	3	20.67	1.55E–49
start_lost	64	54	2	34	1	27	1.08E–17
missense_variant	2221	159	89	100	43	1.79	1.48E–30
stop_gained	470	154	26	97	12	5.92	5.15E–57

LoF, loss-of-function; OR, odds ratio

61% (97/159) of the SPDVs were in *NF1* gene and were classified as pathogenic or likely pathogenic according to the American College of Medical Genetics and Genomics and the Association of Molecular Pathology published standards for the Interpretation of Sequence Variants [55], as well as the updated ClinGen guidance (https://clinicalgenome.org/working-groups/sequence-variant-interpretation/). In 55 newly enrolled CPT cases, 63 rare LoFs or missense variants were identified (Supplementary Table 2). In these patients, nine of them had SPDVs in two genes: *NF1* and another ossification-related gene (Supplementary Table 2). For instance, patients M2801 and M4041 had SPDVs in both *NF1* and *GLI3*. Patients M3748, M4108 and 56A had SPDVs in both *NF1* and *MRC2*. Patients M4363, 52A and 72A had SPDVs in both *NF1* and *PTH1R*. M3722 had SPDVs in both *NF1* and *RYR1* (Supplementary Tables 1 and 2).

### Gene burden test of rare SPDVs

The gene burden test identified 102 genes significantly affected by the rare SPDVs with P values less than 0.05. Among these, 28 genes (27.45%) were significantly represented in the 1239 genes of the osteocyte transcriptome signature which control bone structure and function (hypergeometric test, P = 2.14e-9) [56]. We examined the expressions of the 102 genes in the osteocyte transcriptome of mice tibia and removed half of the genes with low expressions, using a mean FPKM cutoff of 8.24 [56]. GO biological processes enrichment of the remaining 51 genes revealed significant enrichment in only two processes: homophilic cell adhesion via plasma membrane adhesion molecules (GO:0007156, 10/167, Benjamini–Hochberg adjusted P = 1e-8.248), and ossification (GO:0001503, 7/285, Benjamini–Hochberg adjusted P = 1e-2.507). However, the enrichment in homophilic cell adhesion was deemed a false positive due to overlapping intervals in the protocadherin gamma gene cluster members (*PCDHGB2*, *PCDHGB3*, *PCDHGB4*, *PCDHGB5*, *PCDHGB6*, *PCDHGA5*, *PCDHGA6*, *PCDHGA7*, *PCDHGA8* and *PCDHGA9*).

**Fig. 1:**
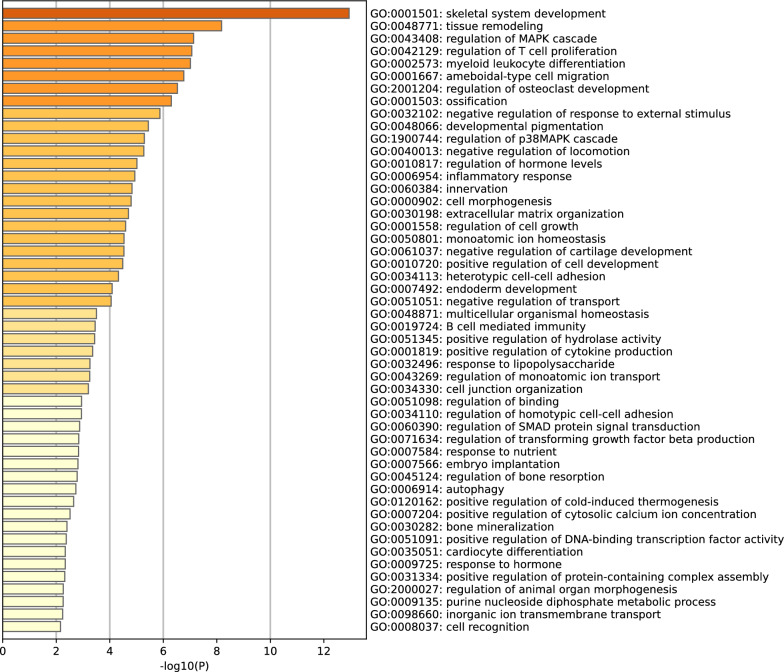
Enrichment of genes with SPDVs in GO analysis. The genes with SPDVs in CPT cases significantly enriched in biological processes of Gene Ontology databases with the Bonferroni corrected P = 0.05/100 = 5e-4

**Fig. 2:**
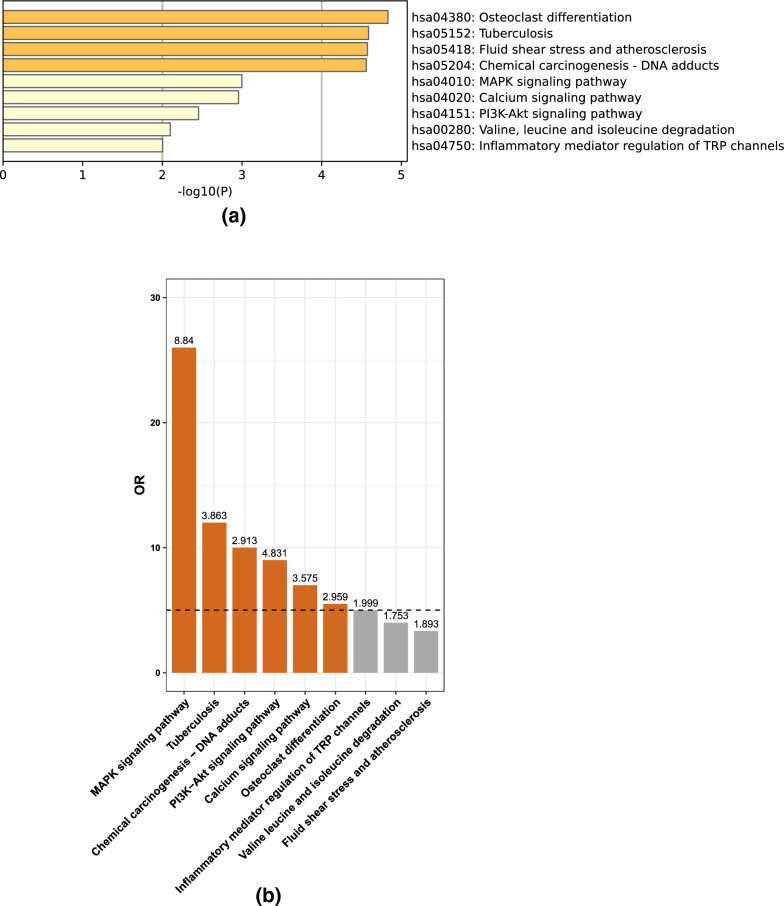
The KEGG pathway enrichment analysis and the LoF excesses of the transcriptome high expression (THE) gene set with rare genomic LoF variations. **a** KEGG enrichment of THE carrying LoF mutations. X-axis indicates the -log(P) in which P is the probability that the genes are enriched in the pathway. A threshold of -log(P) = 2 corresponds to a P value of 0.01. **b** LoF excesses of the significant KEGG pathways enriched by THE. The x-axis lists the pathways from KEGG database and Y-axis indicates the odds ratio that the LoF mutations enrich in the pathway between 159 CPT cases and 208 controls. The numbers above the bars represent the -log(P) values, with a threshold of -log(P) = 2, corresponding to a P value of 0.01

Ultimately, 41 genes carrying 191 SPDVs in 112 CPT cases (Fig. 1) and ossification (GO:0001503) emerged as the only rational CPT-related biological process, which involved seven genes (*GLI3, NF1, NPR2, PTH1R, RYR1, MRC2* and *ITGA11*) with 63 SPDVs in 55 CPT cases (Supplementary Table 2). Consistent with the previous research, we also found that *NF1*, which carried 65 SPDVs in 66 cases (41.5%), was significantly associated with CPT (P = 2.63e-23). Interestingly, osteoblast differentiation (GO:0001649), a child process of the ossification (GO:0001503), is significantly enriched by the gene set of *NF1, GLI3, PTH1R, MRC2* and *ITGA11* (Benjamini–Hochberg adjusted P = 0.018) (Fig. 2a, b). This provides a more detailed biological process to explain the etiology of CPT.

### Seven candidate genes may contribute to CPT in ossification by osteoblast differentiation

Among the genes with rare SPDVs in the 159 cases, *GLI3, NF1, NPR2, PTH1R, RYR1, MRC2* and *ITGA11* were associated with the ossification biological process. Ossification is defined as the formation of bone or of a bony substance, or the conversion of fibrous tissue or of cartilage into bone or a bony substance. Ossification regulates osteocyte network formation and function in human skeletal disease [56]. Clinical phenotypes of CPT indicate a defect in ossification.

Among the seven genes, *NF1, GLI3, PTH1R, MRC2* and *ITGA11* are related to osteoblast differentiation, an essential process in ossification. Osteoblast differentiation is defined as the transformation of relatively unspecialized cells into osteoblasts, which are mesodermal or neural crest cells responsible for forming bone tissue [57, 58]. Osteoclasts and osteoblasts play key roles in bone remodeling, where new bone tissue replaces old bone through bone-forming osteoblasts and bone-resorbing osteoclasts [59]. The enrichment of SPDVs in the biological process of osteoblast differentiation under ossification provides a more detailed understanding of how *NF1* contributes to CPT.

*GLI3*, a crucial regulator in the Hedgehog pathway, may interact with *NF1*, affecting the RAS-MAPK signaling pathway. This interaction ensures balanced regulation of both pathways during osteoblast differentiation. *PTH1R* participates in parathyroid hormone signaling, affecting bone cell metabolism. During osteoblast differentiation, *PTH1R* activities are modulated by *GLI3* and *NF1*, ensuring appropriate hormone signaling for proper cell differentiation. *MRC2* and *ITGA11* are involved in interactions with the extracellular matrix, potentially influencing the interaction between bone cells and the matrix, thereby regulating bone matrix formation.

**Fig. 3:**
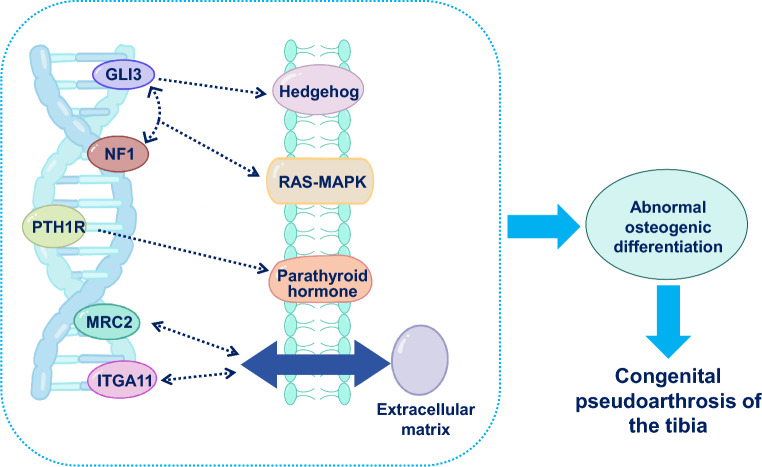
A schematic illustrating genes and their interplay that may be involved in the development of tibial pseudoarthrosis

In summary, the interplay of *GLI3, NF1, PTH1R, MRC2,* and *ITGA11* in osteoblast differentiation encompasses diverse collaborative mechanisms across signaling pathways, hormonal regulations, and extracellular matrix interactions (Fig. 3). This coordinated regulation is crucial for maintaining normal bone cell differentiation and overall skeletal health.

As for the other two genes, *RYR1* mutations may impact calcium signaling, affecting cellular processes involved in bone formation and ossification. While *RYR1* mutations are primarily associated with skeletal muscle disorders, their potential roles in bone disorders require further investigation. *NPR2* mutations may affect the natriuretic peptide signaling pathway, disrupting the regulation of bone growth and ossification, leading to skeletal dysplasia characterized by abnormal bone development and ossification.

Overall, these results highlight that the integrated coordination of cellular signaling, hormonal regulation of bone cells, and extracellular matrix formation is essential for maintaining normal bone cell differentiation. This collaboration ensures normal bone cell differentiation and prevents skeletal developmental anomalies or bone-related disorders.

## Discussion

CPT is a rare bone disorder primarily affecting newborns, characterized by challenging complications such as refracture, tibial non-union, and failed surgery [60]. The underlying pathophysiology involves excessive osteoclast activity caused by the fibrous hamartoma surrounding the bone and impaired osteogenesis and bone morphogenetic protein function, resulting in frequent tibial fractures, bone atrophy, and compromised bone remodeling [61]. WES of 159 CPT cases revealed that the rare LoF variations in osteocyte-related pathways accounted for 70.4% of the CPT cohort. Gene burden test indicated that *NF1, GLI3, NPR2, PTH1R, RYR1, MRC2* and *ITGA11* were related to the ossification biological process, with *NF1, GLI3, PTH1R, MRC2* and *ITGA11* being significantly enriched in osteoblast differentiation. Pathogenic variants in *NF1* were identified in 61% of the patients, with nine patients having LoF variants in both *NF1* and another ossification-related gene.

*NF1* mutation, with the prevalence of 1/3,000 [62], is linked to CPT in many cases and is known to contribute to anterolateral bowing of the tibia, leading to pathologic fracture. *NF1* encodes neurofibromin, a ubiquitous protein expressed in osteoblasts, osteoclasts, chondrocytes, fibroblasts and endothelial cells. Neurofibromin negatively regulates Ras activity, impacting cellular proliferation and differentiation. *NF1* plays a crucial role in preventing bony bridging and endochondral bone repair by inhibiting the invasion and increasing proliferation of fibrous lesion cells, thereby contributing to bone development, homeostasis, and repair in *NF1*-related deficiencies [63]. In 2006, Stevenson proposed the theory that a secondary inactivation of the *NF1* gene in tibial lesion tissue may contribute to CPT pathogenesis, involving the neurofibromin-mediated RAS signaling pathway [11]. Sang Min Lee found loss of heterozygosity in the *NF1* gene in pathological tissues of 4 out of 16 CPT cases, suggesting that double inactivation of the *NF1* gene and the cloning of pathological tissues may contribute to CPT pathogenesis [8].

While *NF1* is implicated in CPT, other factors like intrauterine trauma and metabolic disturbances at birth also play a role [64]. Recent studies have identified several biological processes that are associated with CPT, such as skeletal system development, tissue morphogenesis, cell proliferation and differentiation [15, 18, 65]. These findings suggest the involvement of intricate molecular processes in which other genes, in conjunction with *NF1*, may contribute to CPT.

This study found nine patients harboring other ossification-related genes in addition to *NF1*. For example, two patients had rare variants in *GLI3,* which encodes a protein belonging to the C2H2-type zinc finger proteins subclass of the Gli family. Mutations in this gene are associated with several diseases, including Greig cephalopolysyndactyly syndrome (OMIM: 175,700), Pallister-Hall syndrome (OMIM: 146,510), preaxial polydactyly type IV (OMIM: 174,700), and postaxial polydactyly types A1 and B (OMIM: 174,200). *GLI3* plays a crucial role in limb development as a transcriptional activator and repressor of the sonic hedgehog pathway. The full-length *GLI3* form (*GLI3FL*), after phosphorylation and nuclear translocation, acts as an activator (*GLI3A*), while *GLI3R*, its C-terminally truncated form, acts as a repressor. A proper balance between *GLI3A* and *GLI3R*, rather than the gradient of either, specifies limb digit number and identity. Three patients also had rare variants in both *NF1* and *PTH1R*. *PTH1R* is a protein-coding gene. Diseases associated with *PTH1R* include chondrodysplasia, blomstrand type (OMIM: 215,045), metaphyseal chondrodysplasia, Jansen type (OMIM: 156,400), Eiken syndrome (OMIM: 600,002) and primary failure of tooth eruption (OMIM: 125,350). Among its related pathways are endochondral ossification with skeletal dysplasias and G protein-coupled receptor downstream signaling. Another three patients also had rare variants in both *NF1* and *MRC2*. *MRC2* is a recycling endocytic receptor that functions in cell motility and remodeling of the extracellular matrix by promoting cell migration and collagen uptake for intracellular degradation [66]. One patient also had rare variants in both *NF1* and *RYR1. RYR1* encodes a ryanodine receptor found in skeletal muscle. The encoded protein functions as a calcium release channel in the sarcoplasmic reticulum and also facilitates the connection between the sarcoplasmic reticulum and transverse tubule. Its role extends to normal embryonic development of muscle fibers, skeletal muscle, normal heart morphogenesis, and skin development. Additionally, it plays a role in embryonic ossification, as evidenced by delayed skeletal ossification in neonates with homozygous mutations [67]. The precise mechanisms underlying these effects have not yet been elucidated. Further investigation is required to determine whether these genes act as modifier genes coordinating with *NF1* in contributing to CPT pathogenesis.

Some CPT cases only exhibited rare variants in a single gene, such as *NPR2* and *ITGA11.* Diseases associated with *NPR2* include acromesomelic dysplasia 1 (OMIM: 602,875), epiphyseal chondrodysplasia (OMIM: 615,923) and short stature with nonspecific skeletal abnormalities (OMIM: 616,255). *ITGA11* (integrin α11) is expressed by human bone marrow stromal cells, correlating with osteogenic potential in culture [68]. It also binds osteolectin with nanomolar affinity and is essential for the osteogenic response to osteolectin in humans [69, 70]. The roles of these genes in CPT remain unclear.

Several limitations must be acknowledged when interpreting the findings of our study. In this study, we only studied sporadic CPT cases without verifying whether the identified variants were de novo or inherited. Considering the extremely low prevalence of CPT, de novo mutations would serve as more accurate genomic markers for the genetic diagnosis of CPT within our current pathway framework. Furthermore, the biological processes and functions of the identified rare variants in genes other than *NF1* require further investigation to understand their role in CPT.

In summary, our findings highlight the crucial role of rare genomic LoF variations in genes associated with osteoclast differentiation and regulation pathways in the pathological process of CPT. This provides a new framework for understanding the regulation of biological processes by genetic variations in CPT pathology.

## Supplementary Information

Supplementary material 1: Supplementary Table 1: Clinical phenotypes and genotypes with SPDVs in 159 CPT cases.

Supplementary material 2: Supplementary Table 2. The identified 63 rare LoF or missense variant in 55 CPT cases in this study.

## Data Availability

The authors declare that all data supporting the findings of this study are available within the article and its Additional files or by contacting the corresponding author upon reasonable request.

## Abbreviations

CPT: Congenital pseudarthrosis of the tibia
LoF: Loss of function
NF1: Neurofibromatosis type 1
PDV: Protein-damaging variations
SPDVs: Singleton PDVs
WES: Whole exome sequencing
